# A Cretaceous sap beetle with specialized mandibles (Coleoptera : Nitidulidae)

**DOI:** 10.1098/rsos.241761

**Published:** 2025-02-26

**Authors:** Qian Zhao, Michael S. Engel, Diying Huang, Chenyang Cai

**Affiliations:** ^1^ State Key Laboratory of Palaeobiology and Stratigraphy, Center for Excellence in Life and Palaeoenvironment, Nanjing Institute of Geology and Palaeontology, Chinese Academy of Sciences, Nanjing 210008, People’s Republic of China; ^2^ University of Chinese Academy of Sciences, Beijing 100049, People’s Republic of China; ^3^ Division of Invertebrate Zoology, American Museum of Natural History, 200 Central Park West, New York, NY 10024-5192, USA; ^4^ Facultad de Ciencias Biológicas, Universidad Nacional Mayor de San Marcos, Lima 15081, Peru; ^5^ Departamento de Entomología, Museo de Historia Natural, Universidad Nacional Mayor de San Marcos, Lima 15072, Peru

**Keywords:** taxonomy, palaeoentomology, sexual dimorphism, pollination

## Abstract

Sap beetles (Nitidulidae) are species-rich, highly diverse, widely distributed and exhibit varied food habits. However, studies on nitidulids in Kachin amber are scarce, particularly those involving nitidulids with specialized mandibles. Here, we report a new genus and species of Nitidulidae, *Vetunitidula mandibulata* gen. et sp. nov., from mid-Cretaceous Kachin amber (approx. 99 Ma). This species is characterized by distinctly enlarged mandibles and a loose three-article antennal club, suggesting it as a stem-group nitidulid. The enlarged mandibles may be a manifestation of sexual dimorphism, as in some extant species. Together with previous studies of fossil nitidulids, our discovery highlights the remarkable diversity and morphological disparity of sap beetles during the late Mesozoic.

## 1. Introduction

Nitidulidae, commonly known as sap beetles, are the most diverse family within the superfamily Nitiduloidea, comprising over 350 extant genera and nearly 4500 species [1]. Extant Nitidulidae are divided into 11 subfamilies: Calonecrinae Kirejtshuk, Carpophilinae Erichson, Amphicrossinae Kirejtshuk, Meligethinae Thomson, Epuraeinae Kirejtshuk, Nitidulinae Latreille, Cillaeinae Kirejtshuk and Audisio, Maynipeplinae Kirejtshuk, Cryptarchinae Thomson, Cybocephalinae Jacquelin duVal, and Prometopinae Böving and Craighead [1–3]. Nitidulid beetles are globally distributed, with higher abundance in tropical and Holarctic regions [1]. Their feeding habits are diverse, including mycophagy, predation, saprophagy, necrophagy, anthophagy, frugivory and tree fluid-feeding [1,4]. While most nitidulids feed on fungi or sap, Meligethinae and some species within Epuraeinae, Carpophilinae and Nitidulinae are primarily anthophagous [4].

Kachin amber is a crucial palaeontological resource, renowned for its exceptional preservation of mid-Cretaceous biodiversity [3,5–7]. This amber encapsulates a wide array of organisms, including bryophytes, flowering plants, gastropods, arachnids, vertebrates and a diverse range of insects, offering invaluable insights into an ancient ecosystem and the history of terrestrial life on Earth during the mid-Cretaceous [7]. To date, approximately 40 genera and over 65 species of Nitidulidae have been reported as fossils [8–11], although only 14 species are known from the Mesozoic. Among these fossils, five genera and nine species are known as impression fossils, including five species from the Jurassic: *Nitidulites argoviensis* Heer, 1865, *Petrorophus truncatus* Heer in Heer and Escher, 1852, *Strongylites lavigatus* Heer, 1865, *Strongylites morio* Heer, 1865 and *Strongylites stygicus* Heer, 1865, and four species from the Cretaceous: *Crepuraea archaica* Kirejtshuk, 1990, *Crepuraea explanata* Kirejtshuk, 1990, *Crepuraea zherichini* Kirejtshuk and Ponomarenko, 1990 and *Cyllolithus mirandus* Kirejtshuk, 1990 [12–14]. The remaining five species are preserved in Kachin amber: *Sorodites angustipes* Kirejtshuk, 2018, *Protonitidula neli* Zhao *et al*., 2022, *Phenolia* (*Palaeoronia*) *haoranae* Kirejtshuk and Jenkins Shaw, 2023, *Spinanitidula nigroflavo* and *Cretabaltoraea volsella* Peris, Jelínek & Audisio, 2024 [8,10,11,15,16]. In addition to the fossil species mentioned above, previous species in Kachin amber that were initially attributed to the Kateretidae have been reclassified as belonging to the extinct subfamily Apophisandrinae within Nitidulidae based on morphological analysis [17]. However, as precise phylogenetic relationships between Kateretidae and Nitidulidae remain unresolved, we remain cautious about fully endorsing this reclassification at the family level for the previously reported kateretids. Nonetheless, we acknowledge that the newly described genera and species assigned to this subfamily, such as *Diopsiretes corniger* Peris, Jelínek & Audisio, 2024 and *Cornuturetes elaphus* Peris, Jelínek & Audisio, 2024, are correctly placed within Nitidulidae [17]. Here, we report a new genus and species of Nitidulidae in Kachin amber, exhibiting robust and enlarged mandibles, representing the first fossil record of Mesozoic nitidulid with carinae on specialized mandibles. Moreover, the close proximity of the sap beetle to a stenurothripid thrips, often considered gymnosperm pollinators in the Mesozoic, within the same piece of amber enhances the likelihood of shared floral associations and potential ecological interactions between these insects and gymnosperms.

## 2. Material and methods

The unique specimen (accession number, NIGP203942) is permanently housed in the Nanjing Institute of Geology and Palaeontology, Chinese Academy of Sciences, Nanjing, China. The amber piece was ground with sandpapers of different grit sizes and ultimately polished with diatomite mud for photography [18]. Photographs were taken using a Zeiss Stereo Discovery V16 microscope system with incident and transmitted light, and a Zeiss LSM 710 confocal laser scanning microscope (CLSM) with an attached digital camera [19,20]. Helicon Focus 7.0.2, Adobe Photoshop CC 2019 and Adobe Illustrator 2020 were used for image and figure plate composition.

The Kachin amber specimen was sourced from an amber mine near the Noije Bum Hill summit site, 20 km southwest of Tanai, in the Hukawng Valley, Kachin Province, northern Myanmar [21,22]. Zircon dating of wall rocks and palaeontological evidence suggests that the age of Kachin amber is mid-Cretaceous, from the latest Albian to the earliest Cenomanian (approx. 98.79 ± 0.62 Ma) [23]. The nomenclatural acts established herein are registered under LSIDurn:lsid:zoobank.org:pub:8C88BB0E-B1B2-4BCA-8DAF-EDE381AEAE56.

## 3. Results

### 3.1. Systematic palaeontology

Order: Coleoptera Linnaeus, 1758

Superfamily: Nitiduloidea Latreille, 1802
Family: Nitidulidae Latreille, 1802
Genus: *Vetunitidula* gen. nov. (LSIDurn:lsid:zoobank.org:act:6BA19326-E441-439C-BA1F-DFF19312A36B)

(ⅰ) Type species

*Vetunitidula mandibulata* sp. nov.

(ⅱ) Etymology

The generic name is a combination of the Latin adjective *vetus*, meaning, ‘old’, and the generic name *Nitidula* Fabricius; the name is feminine in gender.

(ⅲ) Diagnosis

Body elongate, dorsally glabrous. Head rectangular, with distinct occipital ridge. Clypeus transverse, labrum distinct. Mandibles large, straight, with distinct medial (mediolongitudinal) carina (complete on left mandible, incomplete on right mandible), with at least two distinct teeth on the mesal edge of each mandible. Antenna concealed, with a loose club composed of three articles. Pronotal and elytral sides explanate horizontally, discs punctured. Prosternal process prolongate, with small lateral projection, procoxal cavities closed incompletely. Elytra truncate, with over two abdominal segments exposed. Meso- and meta-tibiae armed with two longitudinal rows of spines dorsally; two tibial spurs present.

(ⅳ) Description

**Figure 1.**
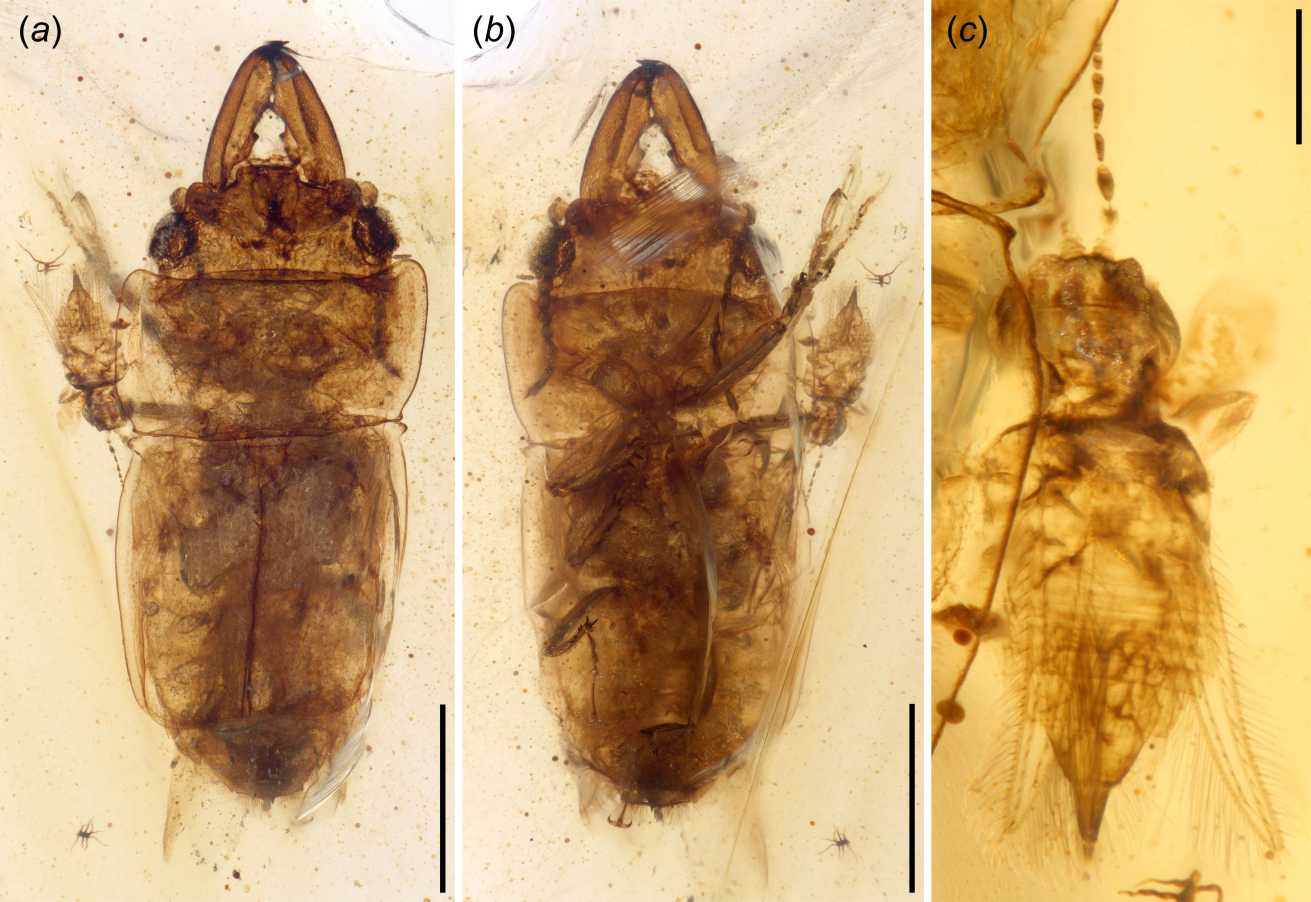
Photomicrographs of *Vetunitidula mandibulata* gen. et sp. nov. (NIGP203942) and a stenurothripid thrips from mid-Cretaceous Kachin amber; under normal reflected light. (*a*) Dorsal view. (*b*) Ventral view. (*c*) Dorsal view of thrips (cf. *Didymothrips abdominalis* Guo *et al.*). Scale bars: 1 mm in (*a, b*) and 0.2 mm in (*c*).

Body elongated and moderately flattened dorsoventrally (figure 1*a,b*). Surface dorsally glabrous but punctured (figure 1*a*).

**Figure 2.**
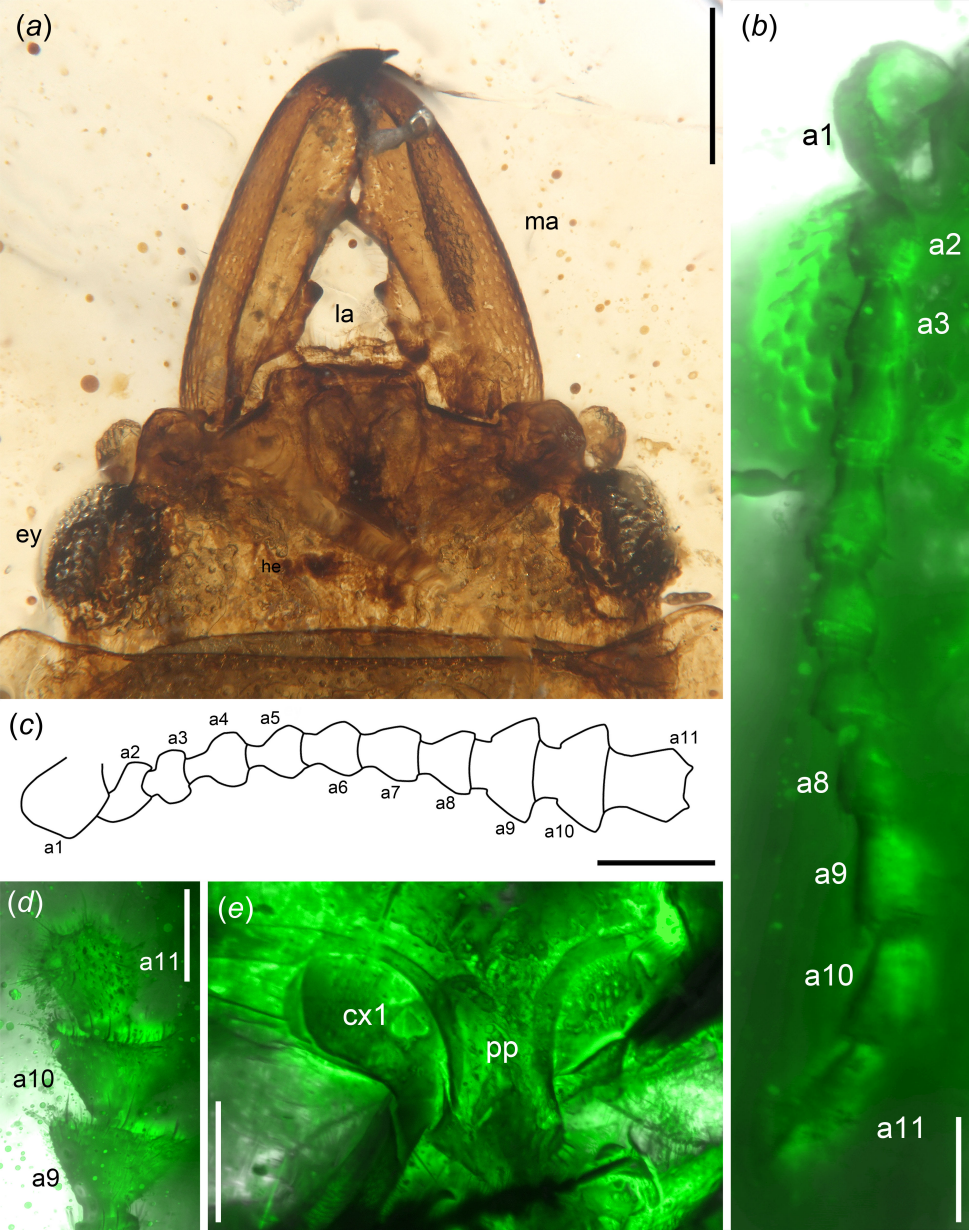
Morphological details of *Vetunitidula mandibulata* gen. et sp. nov. (NIGP203942), viewed with normal reflected light, confocal scanning laser microscopy or by line illustration. (*a*) Dorsal view of head. (*b*) Right antenna, ventral view. (*c*) Line drawing of right antenna, with setae omitted, lateral view. (*d*) Antennal club. (*e*) Ventral view of prothorax. Abbreviations: a1−11, antennomeres I–XI; cx1, procoxa; ey, compound eye; la, labrum; ma, mandible; pp, prosternal process. Scale bars: 0.5 mm in (*a*), 200 μm in (*c, e*) and 100 μm in remainder.

Head prognathous, rectangular, with distinct occipital ridge (figures 1*a* and 2*a*). Mandibles large, straight, punctured, with acute apex; medial (mediolongitudinal) carina distinct and complete dorsally on left mandible, but incomplete on right mandible, and single mandible with at least two distinct teeth on mesal edge (figure 2*a*). Antenna is concealed by anterolateral margins of frons, with loose club composed of three articles (figure 2*a*–*d*). Clypeus transverse, rectangular; labrum distinct, transverse (figure 2*a*).

Pronotum transverse, with prominent and round anterior angles; lateral margin explanate horizontally; pronotal disc punctured, glabrous (figure 1*a*). Prosternum transverse, length slightly shorter than prosternal process; prosternal process prolongate behind procoxae, with lateral projection, apex sparsely setose; procoxal cavities closed incompletely (figures 1*b* and 2*e*).

**Figure 3.**
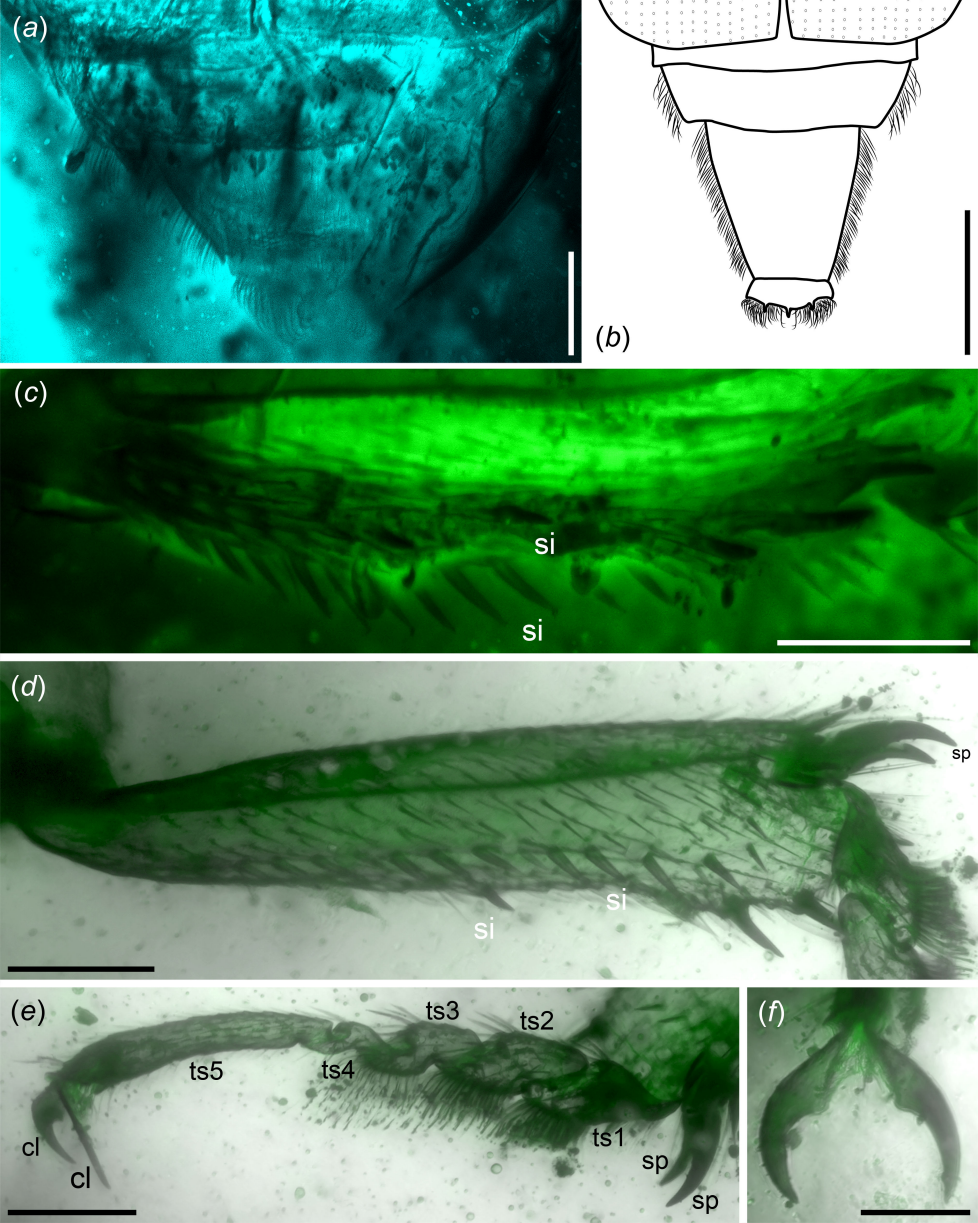
Morphological details of *Vetunitidula mandibulata* gen. et sp. nov. (NIGP203942). All but (*b*) via confocal laser scanning microscopy. (*a*) Dorsal view of exposed abdominal segments; note that the abdomen was bent downwards. (*b*) Reconstructed view of exposed abdominal segments. (*c*) Right mesotibia. (*d*) Right metatibia. (*e*) Right metatarsus. (*f*) Pretarsal claws. Abbreviations: cl, claw; py, pygidium; si, spine; sp, spur; ts1−5, tarsomeres I–V. Scale bars: 500 μm in (*b*), 200 μm in (*a*), 50 μm in (*f*), 100 μm in remainder.

Elytra truncate, quadrate, with over two abdominal segments exposed; anterolateral angles acute horizontally; disc surface seriate-punctate, glabrous; elytral sides explanate horizontally (figures 1*a*, 3*a,b*).

Meso- and meta-tibiae with a longitudinal row of sparse, stout spines and a separate row of dense, long spines on dorsal edge; two tibial spurs present (figure 3*c*,*d*).

*Vetunitidula mandibulata* sp. nov. (LSIDurn:lsid:zoobank.org:act:AFD94918-7761-4FB5-BD41-EF19684F3C38; figures 1–3)

(ⅴ) Etymology

The specific epithet refers to the distinctively large mandibles.

(ⅵ) Diagnosis

As for the genus with the following additional characters: frontoclypeal region is distinctly present. Antennomere 11 is cylindrical. At least one large and blunter tooth and another acute tooth are distinct on the mesal edge of a single mandible. Prosternal process is relatively wide. Triangular mesoscutellar shield is small. Ventrite V with broad, transverse, apical extension with three apical teeth and dense setae.

(ⅶ) Holotype

NIGP203942, tentatively male; the specimen is co-preserved with a Stenurothripidae thrips in close proximity (figure 1)

(ⅷ) Type locality and horizon

Amber mine in the Hukawng Valley, Myitkyina District, Kachin State, Myanmar; earliest Cenomanian (mid-Cretaceous) [24].

(ⅸ) Description

Body length approximately 4.0 mm (measured from apex of mandibles to terminus of pygidium), with greatest width 1.7 mm (measured across midlength of pronotum); colour brownish yellow (diagenetically altered, though, by acidic clearing of integument, may not entirely reflect coloration in life) (figure 1*a*).

Head length 0.6 mm (measured from apex of clypeus to anterior margin of pronotum), width 1.3 mm across compound eyes (figure 1*a*). The anterior edge of the head protruded medially to form a distinct frontoclypeal region (figure 2*a*). Compound eyes protuberant, round, situated at the sides of the head, with short temples (figure 2*a*). Antenna with 11 antennomeres, densely setose; scape twice the length of pedicel; antennomeres II–VIII subequal in length and width, 0.7 × antennomere IX; antennomeres IX and X trapezoidal, subequal in length and width; antennomere XI cylindrical (figure 2*b*‒*d*). Single mandible width 0.2 mm, length 0.8 mm, with one complete mediolongitudinal carina distinct dorsally on the left mandible (incomplete on the right mandible, effaced in the proximal quarter); at least two distinct teeth on the inner edge of each mandible, the first tooth largest, blunter and situated near the base and one acute tooth situated at mid-length (figure 2*a*).

Pronotum widest near the midline, with pronotal length/head length ratio 3 : 2; lateral margin widely explanate (figure 1*a*). Prosternal process is relatively wide, subequal with distance between metacoxae (figure 1*b*). Mesoscutellar shield small, triangular, with rounded apex (figure 1*a*). Meso- and meta-coxae are moderately separated, no wider than the width of the prosternal process (figure 1*b*).

Elytra medial length is equal to combined widths, approximately 1.5 mm; sides moderately explanate (figure 1*a*). Combined exposed abdominal segments are approximately 0.8 mm long (figure 3*a*,*b*). Abdomen with five ventrites, sides wrapped by long, dense setae; ventrite V longest, with broad, transverse, apical extension with three apical teeth and dense setae (figures 2*b* and 3*a,b*).

Femora canaliculate for reception of tibiae (figure 1*b*). Tibiae gradually widened distally, densely setose (figure 3*c*,*d*). Tarsal formula 5−5−5; three basal tarsomeres subequal, shapes simple; tarsomere IV smallest, tarsomeres I–III densely setose ventrally; tarsomere V longest, as long as combined lengths of basal three tarsomeres; pretarsal claws simple (figure 3*e*,*f*).

## 4. Discussion

### 4.1. Systematic position of *Vetunitidula*

The Nitidulidae group in Nitiduloidea is composed of Nitidulidae, Smicripidae and Kateretidae, with Nitidulidae and Kateretidae as the closest sister clades in phylogenetic analysis [3]. *Vetunitidula* is classified within the Nitidulidae group and differs from the closely related Smicripidae by features such as truncate elytra exposing three abdominal segments, 11 antennomeres with a trimerous club, a distinct labrum and a 5−5−5 tarsal formula with tarsomere IV smallest [1,3]. Kateretidae differ from Nitidulidae primarily in the presence of a galea [1,25]. Though the key feature is difficult to observe in this specimen, *Vetunitidula* can be distinguished from Kateretidae by its relatively wide and prolongate prosternal process with lateral projections and long setae on the lateral sides of the pygidium. In contrast, Kateretidae have a short, narrow prosternal process, not dilated posterior to the procoxae and lack long setae on the pygidium [1,25].

The antennal club is a common but important diagnostic trait in all nitiduloid families, regardless of the number of antennomeres or whether the club is loose or compact [3]. While both extant Kateretidae and Smicripidae have a loose trimerous club, the club is compact in extant Nitidulidae [1,25,26]. The trimerous club of *Vetunitidula* is loose and slightly larger than the preceding antennomeres, resembling that of Kateretidae, which is a significant feature to distinguish the genus from extant nitidulids [1,25,26].

Apart from the antennal club, *Vetunitidula* can be distinguished from most subfamilies in Nitidulidae, except Carpophilinae and Cillaeinae, by the following features: transverse head without a distinct neck, truncate elytra exposing over two abdominal segments, explanate and glabrous pronotal and elytral sides, subequal tarsomeres II–IV in length and width, distinct transverse labrum, incompletely closed procoxal cavities, metasternum without a medial sulcus, non-flattened tibiae, a 5−5−5 tarsal formula and simple pretarsal claws [1,2,27].

Carpophilinae and Cillaeinae are the two subfamilies most similar to *Vetunitidula*, as both have truncate elytra exposing at least two abdominal segments [1]. Carpophilinae and Cillaeinae are distinguished by the following character states: Carpophilinae generally lack seriate setae or have them partially, whereas Cillaeinae exhibit prominent seriate–punctate and seriate–setose patterns on the elytra or have glabrous seriate–punctate elytra; the combined length of the exposed abdominal segments is shorter than or subequal to the pronotum in Carpophilinae, whereas it is usually the opposite in Cillaeinae [1,27]. In *Vetunitidula mandibulata*, the combined length of exposed abdominal segments is subequal to pronotal length, which corresponds to species in Carpophilinae, and the elytral disc is glabrous and seriate–punctate in new species, which is consistent with elytra in some Cillaeinae [1,27]. *Vetunitidula mandibulata* seems to be related to the initial transitional species between extant Carpophilinae and Cillaeinae. Based on the above comparison, *V. mandibulata* is a Mesozoic extinct genus, different from all extant subfamilies in Nitidulidae, but the features in this new species could be observed in extant Nitidulidae, except the Kateretidae-like antennal club. The new species is a special species belonging to the Nitidulidae with Kateretidae-like club, given the differentiated manifestation of these features of two families in *V. mandibulata*.

The subfamilial relationships of Nitidulidae remain unclear, but it is relatively certain that Cryptarchinae is the earliest diverging subfamily [4]. Cryptarchinae is relatively primitive in having an incompletely closed procoxal cavity and a simple apical margin of the prosternal process, which is similar to that of *V. mandibulata* [1]. However, the absence of a subantennal groove and a loosely three-segmented antennal club in *V. mandibulata* are ancestral characters and are close to those of Kateretidae [2]. There are mid-Cretaceous nitidulids with compact antennal clubs found in Kachin amber, such as *Sorodites angustipes*, *Phenolia* (*Palaeoronia*) *haoranae* and *Spinanitidula nigroflavo* [8,10,11]. As such, Nitidulidae probably consisted of two major groups: one group shared features similar to Kateretidae, including *V. mandibulata*, *Protonitidula neli*, *Cretabaltoraea volsella*, *Diopsiretes corniger* and *Cornuturetes elaphus* [15–17]; and the other group possessed characters closely resembling those of extant sap beetles, distinctly different from Kateretidae. The latter group may have evolved into the present-day extant Nitidulidae, while the former lineage probably became extinct during subsequent evolution. Accordingly, *Vetunitidula* probably represents a stem-group lineage of Nitidulidae, retaining some symplesiomorphic characters with Kateretidae.

There are 16 Mesozoic species of Nitidulidae, including nine compression fossil species and seven in Kachin amber [8,10–17]. Due to poor preservation, comparing *Vetunitidula* with compression fossils is challenging. However, the enlarged mandibles of *Vetunitidula* make it easily distinguishable [12–14]. Among the amber fossils from northern Myanmar, *Vetunitidula* resembles *Protonitidula* and *Cretabaltoraea*, and differs from the other five species by its prognathous head, specialized mandibles, loose antennal club, absence of horns on the head, truncate elytra exposing three abdominal segments and meso- and meta-tibiae armed with two rows of spines [8,10,11,15–17]. *Vetunitidula* is characterized by over two exposed abdominal segments, absence of jugular projections, nearly parallel lateral sides of the prosternal process with small lateral projections posteriorly, subequal tibial spurs and a pygidium formed of an apical extension with three teeth and dense, long setae. In contrast, *Protonitidula* displays only a partially exposed pygidium, several small teeth on the last sternite, jugular projections, unequal tibial spurs and distinctly curved lateral sides of the prosternal process with prominent lateral projections [15]. Additionally, the differences between the newly described genus and *Cretabaltoraea* primarily involve the number of exposed abdominal segments, the morphology of the labrum and the specialized mandibles, which currently distinguish these two fossil genera [16].

### 4.2. Specialized mandibles

The most striking feature of *Vetunitidula* is its enlarged, straight mandibles. These mandibles are strong, with a medial carina on the dorsal side of each mandible (complete on the left mandible, incomplete on the right mandible), and their mesal margins are asymmetrical, with at least two distinct teeth. Nitidulids with such specialized mandibles are rare in the fossil record, especially in the Mesozoic period, probably due to the incompleteness of fossil preservation and imperfect fossil record. *Cretabaltoraea* is the only Mesozoic fossil record reported by other researchers. Nevertheless, the mandibles in *Cretabaltoraea* are simple, without carinae and tooth [16]. Mandibles in *Vetunitidula* are significantly different from those in *Cretabaltoraea. Vetunitidula* represents the first fossil record of nitidulids with carinae on enlarged mandibles.

Despite the diverse feeding habits of extant Nitidulidae, the morphology of the mandibles of *Vetunitidula* provides valuable clues for exploring their function. In Nitidulidae, mandible specialization does not seem directly related to feeding habits. Extant species with enlarged mandibles occur in Nitidulinae, Cryptarchinae and Cillaeinae, and their feeding habits are diverse and complex [1,4]. Among reported nitidulids with specialized mandibles, *Glischrochilus parvipustulatus* (Kolbe, 1886), *Glischrochilus japonius* (Motschulsky, 1858) (Cryptarchinae), *Prometopia sexmaculata* (Say, 1825) (Prometopinae) and some species of *Platychora* Erichson (Prometopinae) have been found under bark or on tree sap, suggesting they probably feed subcortically on tree sap [28–30]. These species have relatively simple but stout mandibles with one pair of teeth near the apex (or medially in some species) [28–30]. In contrast, some species in *Psilotus* Fischer von Waldheim (Nitidulinae) possess elongate and slender mandibles with at least four preapical teeth, and these species are likely to feed on fungi under palm sheaths [31]. The mandibles of *Vetunitidula* are straighter than those of male *Glischrochilus*, *Prometopia* and *Platychora* [28–30]. Additionally, the mandibles are stouter, with a mediolongitudinal carina dorsally, and have more obtuse teeth compared with those of *Psilotus* [31]. Consequently, it is challenging to infer the feeding habits of *Vetunitidula*.

The scaling of body parts is crucial to morphological evolution. Typically, traits scale proportionally with body size, making larger adults magnified versions of smaller ones. Departures from this pattern, where traits scale disproportionately, can create dramatically exaggerated features. Such extreme morphologies are often hypothesized to result from sexual selection when asymmetrical between the sexes [32,33]. The possibility that the specialized mandibles in *Vetunitidula* are a form of sexual dimorphism cannot be ignored. Sexual dimorphism usually results from selective pressures acting on one sex, leading to a prominent phenotype in that sex relative to the other [34]. In insects, sexual dimorphism often manifests in variations in body size and shape, highly modified antennae and legs, and strong weapons [1,35–37]. Mandibles lacking sexual dimorphism typically exhibit small, simply curved characteristics [30, fig. 1]. Specialized mandibles are common in male beetles as a form of sexual dimorphism, and such traits are observed in extant Nitidulidae, especially in Nitidulinae, Cryptarchinae and Cillaeinae [1]. For example, males of these species typically possess specialized mandibles, whereas females generally do not. *Librodor japonicus* serves as a representative. In *L. japonicus*, ‘The mandibles of males were larger and were straighter and thicker compared with the smaller, smoothly curved mandible of females’ [38, fig. 1]. Similarly, the mandibles of the *Vetunitidula* specimen described herein are extremely enlarged, straight and thick, resembling those of male *L. japonicus*. It suggests a possible speculation that *Vetunitidula* probably exhibits sexual dimorphism, with its enlarged mandibles a manifestation of such asymmetrical exaggeration between the sexes, even though no female has yet been discovered (in living species, females never have such exaggerated mandibles). The specialized mandibles in these extant beetle species usually indicate that the males are stronger and more resistant to diseases and parasites, and play important roles in male–male competition and attracting females to assert resource ownership [34,38,39].

In Nitidulidae, the specialized mandibles are often allometric, and the mandibles of *Vetunitidula* are probably no exception [38]. For example, in the extant nitidulid *L. japonicus*, the outcome of male–male competition depends on the resources possessed by the male. This ownership status-dependent strategy may maintain intra-sexual dimorphism in smaller males of *L. japonicus* [38,40]. Although there is only one specimen of *Vetunitidula*, such a specialized mandible is rare in fossil specimens. Therefore, the possibility cannot be discarded, that it may be a sexual dimorphic trait and serve as the function described above.

### 4.3. Associated thrips and palaeoecological implications

The holotype of *V. mandibulata* represents the first fossil occurrence of a nitidulid preserved alongside a thrips in the same amber specimen. The presence of a thrips of the family Stenurothripidae (cf. *Didymothrips abdominalis* Guo, Engel, Shih, & Ren) close to the nitidulid (figure 1*c*) suggests possible habits of *V. mandibulata*. Some extant species of thrips play crucial roles as pollinators of angiosperms and gymnosperms [41]. Thrips were reported to pollinate lauralean flowers in the Early Cretaceous [42], but also most thrips are considered gymnosperm pollinators during the Mesozoic, evidenced by co-preserved cycad pollen in Early Cretaceous Spanish amber and mid-Cretaceous Kachin amber [41,43]. The co-preservation of *V. mandibulata* and a stenurothripid thrips in the same amber suggests that these insects probably shared the same microenvironment and perhaps similar ecological associations, such as with cycads, some 99 Ma. This raises the intriguing possibility that *V. mandibulata* might have been a pollen feeder of gymnosperms during the mid-Cretaceous. No pollen was found around the thrips or within this sample of amber, and so such pollinivory for *Vetunitidula* requires further testing as new material is discovered. Nonetheless, given the association, beetle pollinivory as a good hypothesis is worth further exploring.

## 5. Conclusion

Nitidulidae are a diverse family of beetles with high biodiversity, yet reports of nitidulids in Kachin amber are rare. We describe a new genus and species of Nitidulidae characterized by distinctly enlarged mandibles, representing the first fossil record of Mesozoic nitidulids with carinae on specialized mandibles. And, we briefly discuss the possibility of the specialized mandibles being sexual dimorphism, as inferred from comparisons with extant male nitidulids with large mandibles. Additionally, we tentatively propose the hypothesis that co-preservation of thrips in the same amber piece may provide insights into the habitat and ecological function of *V. mandibulata* during the mid-Cretaceous.

## Data Availability

We declare that there is no data in the paper, and the specimen referred in the paper (accession number, NIGP203942) is permanently housed in the Nanjing Institute of Geology and Palaeontology, Chinese Academy of Sciences, Nanjing, China, and we declare that the measurement of specimen is operated by Adobe Photoshop CC 2019. Supplementary material is available online [44].
